# Non-invasive brain stimulation and neuroenhancement

**DOI:** 10.1016/j.cnp.2022.05.002

**Published:** 2022-05-25

**Authors:** Andrea Antal, Bruce Luber, Anna-Katharine Brem, Marom Bikson, Andre R. Brunoni, Roi Cohen Kadosh, Veljko Dubljević, Shirley Fecteau, Florinda Ferreri, Agnes Flöel, Mark Hallett, Roy H. Hamilton, Christoph S. Herrmann, Michal Lavidor, Collen Loo, Caroline Lustenberger, Sergio Machado, Carlo Miniussi, Vera Moliadze, Michael A Nitsche, Simone Rossi, Paolo M. Rossini, Emiliano Santarnecchi, Margitta Seeck, Gregor Thut, Zsolt Turi, Yoshikazu Ugawa, Ganesan Venkatasubramanian, Nicole Wenderoth, Anna Wexler, Ulf Ziemann, Walter Paulus

**Affiliations:** a Department of Neurology, University Medical Center, Göttingen, Germany; b Noninvasive Neuromodulation Unit, Experimental Therapeutics and Pathophysiology Branch, National Institute of Mental Health, Bethesda, MD, USA; c University Hospital of Old Age Psychiatry, University of Bern, Bern, Switzerland; d Department of Old Age Psychiatry, Institute of Psychiatry, Psychology and Neuroscience, King’s College London, London, United Kingdom; e Biomedical Engineering at the City College of New York (CCNY) of the City University of New York (CUNY), NY, USA; f Departamento de Clínica Médica e de Psiquiatria, Faculdade de Medicina da Universidade de São Paulo, São Paulo, Brazil; g Service of Interdisciplinary Neuromodulation (SIN), Laboratory of Neurosciences (LIM-27), Institute of Psychiatry, Hospital das Clínicas da Faculdade de Medicina da USP, São Paulo, Brazil; h School of Psychology, Faculty of Health and Medical Sciences, University of Surrey, Guildford GU2 7XH, UK; i Science, Technology and Society Program, College of Humanities and Social Sciences, North Carolina State University, Raleigh, NC, USA; j Department of Psychiatry and Neurosciences, Faculty of Medicine, Université Laval, CERVO Brain Research Centre, Centre intégré universitaire en santé et services sociaux de la Capitale-Nationale, Quebec City, Quebec, Canada; k Unit of Neurology, Unit of Clinical Neurophysiology, Study Center of Neurodegeneration (CESNE), Department of Neuroscience, University of Padua, Padua, Italy; l Department of Clinical Neurophysiology, Kuopio University Hospital, University of Eastern Finland, Kuopio, Finland; m Department of Neurology, Universitätsmedizin Greifswald, 17475 Greifswald, Germany; n German Centre for Neurodegenerative Diseases (DZNE) Standort Greifswald, 17475 Greifswald, Germany; o Human Motor Control Section, National Institute of Neurological Disorders and Stroke, National Institutes of Health, Bethesda, MD, USA; p Department of Neurology, University of Pennsylvania, Philadelphia, PA, USA; q Experimental Psychology Lab, Department of Psychology, Carl von Ossietzky Universität, Oldenburg, Germany; r Department of Psychology and the Gonda Brain Research Center, Bar Ilan University, Israel; s School of Psychiatry and Black Dog Institute, University of New South Wales; The George Institute; Sydney, Australia; t Neural Control of Movement Lab, Institute of Human Movement Sciences and Sport, Department of Health Sciences and Technology, ETH Zurich, 8092 Zurich, Switzerland; u Department of Sports Methods and Techniques, Federal University of Santa Maria, Santa Maria, Brazil; v Laboratory of Physical Activity Neuroscience, Neurodiversity Institute, Queimados-RJ, Brazil; w Center for Mind/Brain Sciences – CIMeC and Centre for Medical Sciences - CISMed, University of Trento, Rovereto, Italy; x Institute of Medical Psychology and Medical Sociology, University Medical Center Schleswig Holstein, Kiel University, Kiel, Germany; y Department Psychology and Neurosciences, Leibniz Research Centre for Working Environment and Human Factors at TU, Dortmund, Germany; z Dept. Neurology, University Medical Hospital Bergmannsheil, Bochum, Germany; aa Siena Brain Investigation and Neuromodulation Lab (Si-BIN Lab), Unit of Neurology and Clinical Neurophysiology, Department of Medicine, Surgery and Neuroscience, University of Siena, Italy; ab Department of Neuroscience and Neurorehabilitation, Brain Connectivity Lab, IRCCS-San Raffaele-Pisana, Rome, Italy; ac Precision Neuroscience and Neuromodulation Program, Gordon Center for Medical Imaging, Massachusetts General Hospital, Harvard Medical School, Boston, MA, USA; ad Department of Clinical Neurosciences, Hôpitaux Universitaires de Genève, Switzerland; ae Centre for Cognitive Neuroimaging, School of Psychology and Neuroscience, EEG & Epolepsy Unit, University of Glasgow, United Kingdom; af Department of Neuroanatomy, Institute of Anatomy and Cell Biology, Faculty of Medicine, University of Freiburg, Freiburg, Germany; ag Department of Human Neurophysiology, Fukushima Medical University, Fukushima, Japan; ah Department of Psychiatry, National Institute of Mental Health and Neurosciences, Bengaluru, India; ai Future Health Technologies, Singapore-ETH Centre, Campus for Research Excellence And Technological Enterprise (CREATE), Singapore; aj Department of Medical Ethics and Health Policy, University of Pennsylvania Perelman School of Medicine, Philadelphia, PA, USA; ak Department of Neurology and Stroke, University of Tübingen, Germany; al Hertie Institute for Clinical Brain Research, University of Tübingen, Germany; am Department of of Neurology, Ludwig Maximilians University Munich, Germany

**Keywords:** AD, Alzheimer’s Disease, BDNF, brain derived neurotrophic factor, DARPA, Defense Advanced Research Projects Agency, DIY, Do-It-Yourself, DLPFC, dorsolateral prefrontal cortex, EEG, electroencephalography, EMG, electromyography, FCC, Federal Communications Commission, FDA, (U.S.) Food and Drug Administration, IFCN, International Federation of Clinical Neurophysiology, LTD, long-term depression, LTP, long-term potentiation, MCI, mild cognitive impairment, MDD, Medical Device Directive, MDR, Medical Device Regulation, MEP, motor evoked potential, MRI, magnetic resonance imaging, NIBS, noninvasive brain stimulation, OTC, Over-The-Counter, PAS, paired associative stimulation, PET, positron emission tomography, PPC, posterior parietal cortex, QPS, quadripulse stimulation, RMT, resting motor threshold, rTMS, repetitive transcranial magnetic stimulation, SAE, serious adverse event, SMA, supplementary motor cortex, tACS, transcranial alternating current stimulation, TBS, theta-burst stimulation, tDCS, transcranial direct current stimulation, tES, transcranial electric stimulation, TMS, transcranial magnetic stimulation, tRNS, transcranial random noise stimulation, Neuroenhancement, Cognitive enhancement, Transcranial brain stimulation, tDCS, tACS, Home-stimulation, DIY stimulation

## Abstract

Attempts to enhance human memory and learning ability have a long tradition in science. This topic has recently gained substantial attention because of the increasing percentage of older individuals worldwide and the predicted rise of age-associated cognitive decline in brain functions. Transcranial brain stimulation methods, such as transcranial magnetic (TMS) and transcranial electric (tES) stimulation, have been extensively used in an effort to improve cognitive functions in humans.

Here we summarize the available data on low-intensity tES for this purpose, in comparison to repetitive TMS and some pharmacological agents, such as caffeine and nicotine. There is no single area in the brain stimulation field in which only positive outcomes have been reported. For self-directed tES devices, how to restrict variability with regard to efficacy is an essential aspect of device design and function. As with any technique, reproducible outcomes depend on the equipment and how well this is matched to the experience and skill of the operator. For self-administered non-invasive brain stimulation, this requires device designs that rigorously incorporate human operator factors. The wide parameter space of non-invasive brain stimulation, including dose (e.g., duration, intensity (current density), number of repetitions), inclusion/exclusion (e.g., subject’s age), and homeostatic effects, administration of tasks before and during stimulation, and, most importantly, placebo or nocebo effects, have to be taken into account. The outcomes of stimulation are expected to depend on these parameters and should be strictly controlled. The consensus among experts is that low-intensity tES is safe as long as tested and accepted protocols (including, for example, dose, inclusion/exclusion) are followed and devices are used which follow established engineering risk-management procedures. Devices and protocols that allow stimulation outside these parameters cannot claim to be “safe” where they are applying stimulation beyond that examined in published studies that also investigated potential side effects.

Brain stimulation devices marketed for consumer use are distinct from medical devices because they do not make medical claims and are therefore not necessarily subject to the same level of regulation as medical devices (i.e., by government agencies tasked with regulating medical devices). Manufacturers must follow ethical and best practices in marketing tES stimulators, including not misleading users by referencing effects from human trials using devices and protocols not similar to theirs.

## Highlights

• The available data frame with a wide parameter space of tES does not allow an overarching protocol recommendation.
• Established engineering risk-management procedures with regard to manufacturing should be followed.
• Consensus among experts is that tES for neuroenhancement is safe as long as tested protocols are followed.

## 1 Introduction

Non-invasive brain stimulation (NIBS) interventions, such as repetitive transcranial magnetic (rTMS) and low intensity electric (tES) stimulation, impact perception, cognition, mood, motor activities, and other brain functions, both in healthy humans and patients (Buch et al., 2017, Ekhtiari et al., 2019, Huang et al., 2009, Lefaucheur et al., 2017, Lefaucheur et al., 2020, Reis et al., 2018). There is active research on if NIBS has the potential to improve (or worsen) performance in competitive environments, school evaluations, athletic competitions up to the level of the Olympics, and in musicianś performance (e.g., Coffman et al., 2014, Luber and Lisanby, 2014). Long-term effects of NIBS when used in uncontrolled ways (e.g., at-home environments) have not been fully explored. Consequently, the available data on these contexts are less often replicated when they are away from experimental settings, although the possible benefits of NIBS in such contexts would have substantial relevance in everyday life.

Neuroenhancement is perceived by the population as a source of promise and concern (Bard et al., 2018). In otherwise healthy subjects, the term ‘neuroenhancement’ can be conceptualized as a variety of interventions and technologies aiming to improve human performance above the subject’s normal performance, beyond what is considered ‘physiologically normal’. Neuroenhancement covers a broad spectrum of interventions, ranging from beverages with high caffeine concentrations to legal drugs such as nicotine, but even to more sophisticated technologies, like NIBS. The latter is the key focus of this paper. A European survey of attitudes relating to science and technology reported that biotechnology interventions (e.g., NIBS) are consistently viewed more favorably when applied therapeutically in patients than for enhancement in healthy individuals (Gaskell et al., 2011). Nevertheless, 56% of the survey participants (mainly researchers and industry partners) approved research aimed at enhancing human performance, with stronger positive regard for tES compared to drugs. In our previously published safety guideline, we put forward the concept “*Neuroenhancement can be defined as any augmentation of core information processing systems in the brain apart from natural training, including the mechanisms underlying perception, attention, conceptualization, memory, reasoning, and motor performance”* (Antal et al., 2017). A broader definition of human enhancement is the one conceptualized in the European SIENNA project (internet page: https://zenodo.org/record/4066557#.YFsC1nkxmUn):”*Human enhancement is a modification aimed at improving human performance and brought about by science-based and/or technology-based interventions in or on the human body”* in various subfields: cognitive (mainly related to intelligence and memory); physical (sport); affective and emotional (well-being); moral (e.g., correcting decision-making behaviour, social norm compliance); longevity (anti-aging), and others. For instance, the potential of NIBS as a cognitive enhancer is described as “…*interventions that improve cognitive abilities, including neuro-stimulation and neuromodulatory techniques,…. These may impact personal identity, for instance, by altering someone’s moods, cognition, behaviour, and basic personality traits.* “ Based on other definitions, the subfields in that document are methodologically defined as *“by reversibility, by relation to the body, by relation to therapy and by field or technique.”* Since ‘enhancement’ aims to improve human performance beyond what is necessary to sustain or restore good health (Juengst and Eric, 1998), a difficult broader definition between pathology and normality is needed, which in turn depends on social and cultural substrates and age. Here, the definition of enhancement can also be different when considering high-performance groups, such as professional athletes, musicians, or other professions that rely heavily on high-end performance. Nevertheless, it should also be stated that only a small number of studies in the NIBS field were designed for ‘meaningful neuroenhancement’, i.e. systematically developing protocols (e.g. which would require titration of the dose of stimulation) that result in relevant effects. Instead, neuroenhancement by NIBS is often the theoretical aim of at-home applications: This ideally should encompass a verified and safe protocol using a stimulator that has been certified according to country-specific (medical product) legal requirements, (e.g., https://www.regulatory-affairs.org/en/development-excellence/news-page/verification-and-validation-of-medical-devices). However, depending on the manufacturer, these requirements are not necessarily being met. Device manufacturers or social media contributors have frequently used scientific results from prior studies to back up and promote their devices or techniques (see Santarnecchi et al., 2013a, Santarnecchi et al., 2013b). Social media users (e.g. http://www.reddit.com) recommend the application of low intensity tES for several purposes, including neuroenhancement, with no or only insufficient differentiation between CE-approved devices and home-made devices consisting of a 9-volt battery with two wires attached to the poles. In 2016, the International Federation of Clinical Neurophysiology (IFCN) warned against the self-(home) application of tES (so-called do-it-yourself or DIY), especially with non-approved devices (https://wfneurology.org/news_events/archived-news/2016-01-25-ifcn). Since then, technical and methodological progress has expanded the diversity of possible stimulation techniques and related protocols.

Here, we aim to provide a timely review of the present scientific knowledge and place it in context with neuronal (cognitive) enhancement by tES in otherwise healthy individuals, also including a comparison with rTMS and well-known neuroenhancing substances such as nicotine and caffeine. Different aspects of neuroenhancement, including possible long-term effects, placebo effects, self-applied and -directed stimulation are discussed. Our main goal is to prevent uncritical and mercantile biased referrals to the scientific framework available in the field. The authors/experts were chosen broadly based on their publication track in the field of NIBS, with experience in neuroenhancement. First, basic aspects and needs were identified on the fields of tES and neuroenhancement, and commented on in several rounds and during several internet meetings. After consensus with regard to the definitions, recommendations etc. was reached, the experts with their specific expertise, were encouraged to support or revise their earlier points in light of the replies of other members of the expert panel. When it was appropriate, the key findings were summed up at the end of each chapter.

## 2 Physical possibilities: rTMS and tES

The two most frequently used non-invasive technologies for modulating cortical activity are repetitive transcranial magnetic stimulation (rTMS) and transcranial direct current stimulation (tDCS). For neuromodulation purposes, a single pulse of TMS needs to be applied repetitively in time, on a specific brain region. Each single TMS pulse is the consequence of a strong (∼8000 A) and brief (∼100–300 µs) electric current passing through a coil inducing a rapidly changing magnetic field. Such a field, unattenuated by the skull, induces, in turn, a secondary electric current in the brain. TMS induced current intensity declines, depending on coil type, proportionately between the square root of the distance from the coil up to 1/diameter; directly affecting cortical regions a few centimeters beneath the coil (Siebner et al., in press). More distant target neurons are influenced transsynaptically (Ardolino et al., 2005, Lang et al., 2005). Changes in neuronal activity according to the parameters of stimulation, such as intensity, polarity and phase of the induced electric field, are modelled with increasing precision breaking them down to individual cell types (Aberra et al., 2020). Simply due to much higher technical demands, combined with higher prices and safety concerns, rTMS presently plays a minor role in neuroenhancement in the domestic context. Its availability in research laboratories may be tempting for the researchers to apply to themselves, although a questionnaire revealed this to be the case in only 8 % of the researchers (Shirota et al., 2014). In addition, the induction of seizures is rare, but still a potential issue in rTMS (Rossi et al., 2021) but not in low-intensity tES (Antal et al., 2017).

TDCS delivers a low-intensity current to the cortex through two or more electrodes placed on the scalp. The neuromodulatory effect relies on the polarity-specific modulation of membrane polarization, which to a first approximation correspond to anodal as excitatory and cathodal as inhibitory (Bindman et al., 1964, Creutzfeldt et al., 1962), although it is important to differentiate between effects mediated by the target electrode and the return electrode. For example, option to enhance targeting of a given region is to use a smaller target electrode (e.g. 4 × 4 cm), warranting a higher current density, and a large return electrode (e.g. 100 cm^2^) (Nitsche et al., 2007). Still smaller electrodes can be used and deployed in arrays (Datta et al., 2009). Beyond electrode size, factors like electrode shape, connector position and conductivities of different electrode materials including saline solutions and electrode gels may influence the outcome (Saturnino et al., 2015).

Production of an aftereffects of both tDCS and rTMS is necessary to produce lasting behavioural changes, as subjects and patients cannot be stimulated continuously for prolonged periods. The strength and duration of an aftereffects have been shown to be cumulative over a series of separate, consecutive, sessions of stimulation (e.g., Reis et al., 2009). The intervals between sessions may be key: for example, repetitive NIBS sessions play a distinct role. Spacing at intervals of several minutes (i.e., 3–30 min) have been explored to obtain greater and more durable changes in neuroplasticity responses than NIBS applied over more prolonged spacing periods (several hours or days), with the latter appearing to induce less stable, and rapidly reversible plasticity (Ghasemian-Shirvan et al., 2022, Goldsworthy et al., 2015, Monte-Silva et al., 2013). It is assumed that long-term potentiation (LTP) and long-term depression (LTD) phenomena at the synaptic level are the physiological mechanisms for durable changes following NIBS (Liebetanz et al., 2002, Nitsche et al., 2003, Sharma et al., 2022, Rossini et al., 2019).

## 3 Comparative enhancement by tDCS, tACS and tRNS

As opposed to the constant current stimulation over time of tDCS, transcranial alternating current (tACS) (Antal et al., 2008) and random noise (tRNS) (Terney et al., 2008) stimulation apply balanced alternating current or paradigms with a DC offset (Marshall et al., 2006). Using a specific stimulation frequency, tACS may modulate the amplitude, frequency and phase of brain oscillations (Antal et al., 2008, Frohlich et al., 2015, Helfrich et al., 2014a, Helfrich et al., 2014b) as does tRNS, which utilizes mostly randomized stimulation frequencies between 100 and 640 Hz (Terney et al., 2008). These modulations can, in turn, alter cognitive functions (Cecere et al., 2015, Clayton et al., 2019, Fertonani et al., 2011, Snowball et al., 2013).

The weaker the stimulation intensity, the weaker the entrainment effect is on endogenous oscillations. Also, with lower intensities the effective frequency range leading to entrainment shrinks, following the so-called Arnold tongue principle (Vosskuhl et al., 2018). tRNS may seem to provide a less complex approach for the self-stimulation (home) user by circumventing the necessity to decide between particular resonance frequencies to be targeted. However, it should not be forgotten that the effect of any given NIBS protocol can depend on individual cognitive and neuronal factors as well as on age (Evans et al., 2018, Frank et al., 2018, Harty and Cohen Kadosh, 2019).

As with tDCS, both tACS and tRNS may fail to produce neural or behavioral effects (Brignani et al., 2013, Fekete et al., 2018, Lafon et al., 2017, Santarnecchi et al., 2016, Schecklmann et al., 2021). A simple assumption when this occurs has been that stimulation intensity might have been too low to modulate neuronal activity in several studies (Antal et al., 2008, Lafon et al., 2017, Vöröslakos et al., 2018). A more complex hypothesis assumes that increasing intensities cause increases in intracellular calcium inflow with lower concentration ranges for achieving inhibition (LTD-like effects) or higher intensities achieving excitation (LTP-like effects (Moliadze et al., 2012, Shorafa et al., 2021)). In this case, a failure to induce inhibition or excitation might be caused by too low or too high intensity or an intensity in the transition zone between inhibition and excitation. Direct comparisons of the enhancement effects of tACS, tRNS, and tDCS are ongoing, and will depend on the type of the task (Berger et al., 2019, Brem et al., 2018, Fertonani et al., 2011, Inukai et al., 2016, Mulquiney et al., 2011, Murphy et al., 2020). However, such results do not indicate that one method is necessarily better than another, as these specific effects may be rooted in the variability of stimulation protocols and the lack of precise knowledge of optimal parameters for each protocol.

## 4 Considering cognitive/behavioral effects by NIBS: Theoretical, neurobiological and physiological framing of neuroenhancement

Given that studies in normal subjects have observed long-lasting effects on cognition in the order of months following a week of low-intensity tES (Snowball et al., 2013, Cappelletti et al., 2013), the multiple-session mode naturally raises ethical questions regarding reversibility, concomitant cognitive adverse effects, and necessity of additional sessions for maintaining desirable effects (Huo et al., 2021). One difficulty in answering such ethical questions is that a theoretical understanding regarding the mechanisms underlying cognitive enhancement using NIBS, which could provide a framework for working out the situations and contexts under that stimulation creates undesirable effects, is only beginning to be formed.

Modifications of functional brain networks will underlie desired effects on cognition but the question has been raised as to whether these may be associated with changes in activity in other networks, either by a rebalancing of resource allocations or by direct network interactions (Brem et al., 2014, Iuculano and Cohen Kadosh, 2013). Such associated changes, which may lead to detrimental and adverse effects, are likely under-recognized, as most NIBS studies can focus on only one or two tasks at a time. These may develop over time and only may become recognizable long after stimulation. In a simple example of a situation, where a performance enhancement, as well as a concomitant performance cost has been produced, 1-Hz rTMS applied to the PPC increased detection accuracy when visual stimuli were presented in the field ipsilateral to the side of stimulation but decreased for stimuli presented in the visual field contralateral to the side of stimulation (Hilgetag et al., 2001). Since 1 Hz rTMS will lower cortical excitability, neural activity was lowered in one part of the visual field: when stimuli occurred in the opposite field, they were more easily perceived (as the competing visual field was inhibited) and performance was enhanced, at the cost of lower performance when stimuli appeared in the inhibited visual field. In a second, less simple, example, following a six-day cognitive training session, combined with anodal tDCS applied over the left dorsolateral prefrontal cortex (dlPFC) and cathodal over the right one, numerical learning was facilitated, but automaticity of performing the mathematical task was impaired when cathodal tDCS was applied over the posterior PPC (Iuculano and Cohen Kadosh, 2013).It is not clear whether the reported negative effect in this case represented a genuine cost accompanying the improvement of one function, or is explained through stimulation of the secondary brain site by the cathodal electrode, inadvertently impairing its function and leading to poorer attention.

A network approach based on a mathematical concept from game theory, i.e., the zero-sum game, attempts to explain findings such as these (Brem et al., 2014). The gains of one set of players at the game’s end are matched by the losses of another set of players, resulting in a net sum of gains and losses of zero. Looking at the brain as a finite system with a limited processing capacity, the zero-sum game could be applied conceptually, such that neural resources applied towards one goal are taken from others. However, while it is worthwhile to consider that any particular stimulation paradigm may be producing unmeasured costs as well as targeted enhancements, there is no clear demonstration of the zero-sum theory in the area of NIBS at present, therefore it is of more theoretical interest.

An alternative to the zero-sum conception is to examine the effects of NIBS enhancement within the context of learning. When using NIBS to facilitate learning, the individual context must be taken into account. For example, there is no doubt that intensified training, e.g., in piano players or mathematicians, leads to top performances with ceiling effects; tDCS here may only worsen performance (Furuya et al., 2014, Krause et al., 2019a, Krause et al., 2019b). Overall, however, it will not come at a cost for a system to inhibit an irrelevant capacity when trying to perform a task, especially as the system is working through the employment of the proper set of capacities to master the task. The “costs” come when external stimulation is performed without carefully considering the dynamics and properties of the systems involved. Simultaneous NIBS/fMRI and NIBS/EEG, or investigation of state-dependence (Silvanto and Pascual-Leone, 2008) and endogenous oscillations (e.g., Zrenner et al., 2018) may identify network nodes and states through which NIBS could effectively enhance processing and others with a tendency towards deterioration. Specifically designed behavioral tests may then determine the “costs.”.

Another approach to understanding the mechanisms for neuroenhancement is to consider the effects of NIBS on rebalancing abnormal activity levels between network nodes or reinforcing those underlying a task (Lang et al., 2005, Sale et al., 2015). NIBS effects are state-dependent, being strongly influenced by the ongoing activity of the targeted region at the time of stimulation (Antal et al., 2007, Bortoletto et al., 2015, Ferreri et al., 2014, Fertonani et al., 2014). Some of the effects of NIBS on network functions may be conveyed by interactions with brain oscillations (e.g. through local entrainment) as brain rhythms have been associated with communication between distant regions, depending on temporally correlated activity or interregional synchrony. In support of this, single or -better- repetitive TMS pulses have been shown to have the ability to phase-reset and align local oscillators in a given cortical region and to increase the amplitude of rhythms that such oscillators are most likely to generate (Paus et al., 2001, Rosanova et al., 2009, Thut et al., 2011). These effects have been considered to be potentially able to transiently modulate specific cognitive operations and behaviors (Ferreri et al., 2014).

Overall, it is clear that a basic understanding of the effects of NIBS at a systems level (i.e., at the level of an individual user of brain stimulation) is only beginning to be worked out. This should provide an additional caveat when considering the use of brain stimulation for enhancement outside of research environments.

## 5 Comparing NIBS to pharmacological neuroenhancement, caffeine and nicotine

Lifestyle-related substances, such as nicotine and caffeine, are associated with neuroenhancement. Data about the performance-enhancing effects of nicotine in healthy humans are limited. Exam performance did not correlate with nicotine consumption, whereas exam-related anxiety did correlate positively (Kusturica et al., 2019). The impact of nicotine on cognitive performance is critically affected by the habitual smoking state. In non-smokers, acute nicotine intake decreased executive functions (Grundey et al., 2015) and also motor learning (Grundey et al., 2017). Habitual smokers showed decreased executive and motor learning under nicotine deprivation compared with non-smokers. These cognitive deficits were, however, reversed by exposure to nicotine (Grundey et al., 2015, Grundey et al., 2017). These results might mean that in healthy, non-smoking humans, nicotine probably has no neuroenhancing but instead performance-worsening effects, while chronic smokers require it for normal functioning. At the level of brain physiology, related effects of nicotine have been shown for tDCS-induced plasticity, and cortical excitability. Here, smokers showed reduced intracortical facilitation, and abolished LTP-like plasticity during nicotine withdrawal, effects that were reversed by nicotine consumption. In non-smokers, nicotine enhanced cortical inhibition, and converted LTP-like into LTD-like plasticity (Grundey et al., 2013, Grundey et al., 2012a, Grundey et al., 2012b). Smoking-state dependent effects of tDCS on cognitive performance have not been explored systematically, but might be relevant based on the impact of smoking state and nicotine on the physiological effects of tDCS.

Caffeine belongs to the methylxanthines and blocks adenosine receptors that mediate predominantly inhibitory effects. This disinhibition caused by adenosine leads to an increase in vigilance and attention (Daubner et al., 2021). Studies in the field with pure caffeine during stimulation are rare. Most data on caffeine effects are based on energy drinks with confounding effects of other ingredients. With regard to mood, caffeine has been reported to have an increasing effect on anxiety, but not on other mood domains (Fiani et al., 2021). With regard to cognition, caffeine was suggested to enhance processing speed, but not general attention (Fiani et al., 2021). The underlying motivations for caffeine intake are mainly concentration and memory enhancement and physical performance improvement (Cappelletti et al., 2015). Caffeine has a positive effect on sustained attention (Repantis et al., 2021). Interestingly, in the context with transcranial stimulation, caffeine mobilizes intracellular calcium (Cappelletti et al., 2015), which in turn plays a distinct role in determining either LTD- or LTP-like aftereffects of tES (Shorafa et al., 2021). Caffeine also appears to improve physical performance in both trained and untrained individuals (Guest et al., 2021).

Increased alertness as measured by pupillometry was reported after a 200 mg dose of caffeine (Zulkifly et al., 2021b). In a direct comparison, tDCS was reported to be a more powerful fatigue countermeasure than caffeine (McIntire et al., 2017). Conversely, caffeine intake only correlated with a practice effect on the grooved pegboard test in conjunction with 20 min tDCS, alone had no effect in this particular setup (Fagerlund et al., 2015). Caffeine has furthermore significant positive effects on both short- and long-term memory, albeit not in children (Fiani et al., 2021). Variability of plasticity effects in tES studies can be accentuated by uncontrolled caffeine consumption. Excitatory plastic aftereffects induced by 1 mA tACS at 140 Hz were turned into inhibition by espresso intake. Interestingly, decaffeinated espresso was not inert, since it reduced the respective stimulation-induced excitability enhancement to baseline. This effect may be due to residual, lower levels of caffeine in decaffeinated coffee or the up to 1,000 chemical components of coffee (Zulkifly et al., 2020). The caffeine content in regular and decaffeinated coffees ranged from 10.9 mg/g to 16.5 mg/g and from 0.34 mg/g to 0.47 mg/g, respectively. 200 mg caffeine also reduced the excitatory effect of quadripulse stimulation with 5 ms intervals in intervention responders (Hanajima et al., 2019).

The impact of caffeine on brain physiology is probably also dependent on habitual intake of the substance. In caffeine naïve subjects, 200 mg caffeine increased motor cortex excitability, and caffeine increased and prolonged the effects of LTP-like plasticity induction via paired associative stimulation with an interstimulus interval of 25 ms (PAS25) (Zulkifly et al., 2021a), but caffeine did not alter 140 Hz tACS-induced excitability changes. In caffeine consumers, the effects were less clear. Interactions were shown between caffeine concentrations, baseline cortical excitability and cortisol levels (Zulkifly et al., 2021b).

In summary, the effects of pharmacological “neuroenhancers” such as nicotine and caffeine are confounding factors in brain stimulation protocols. Ideally, participants should not be mixed with regard to nicotine or caffeine consumption behavior. Simple deprivation of the respective substance likely does not solve the problem, because at least with respect to nicotine, withdrawal has a modulatory effect on stimulation outcomes itself.

## 6 Boosting intelligence and creativity, and the role of training in cognitive enhancement with tES

Intelligence and creativity, cornerstones of civilization, have also been the target of NIBS research. Research to date has yielded heterogeneous results, likely deriving from using a range of different stimulation setups and varied quantification/translation of concepts. “Intelligence” refers to a broad and complex cognitive concept that is associated with a range of life outcomes (Strenze, 2007) and is, therefore, an appealing target. A few studies have combined NIBS with cognitive training and observed an improvement in fluid intelligence after applying tDCS and tRNS compared to tACS (Almquist et al., 2019, Brem et al., 2018). Administering 40 Hz tACS over prefrontal cortex resulted in a shortened response latency (Santarnecchi et al., 2013a, Santarnecchi et al., 2013b, Santarnecchi et al., 2016) particularly in more difficult items when solving fluid intelligence tasks (Neubauer et al., 2017, Pahor and Jaušovec, 2014, Santarnecchi et al., 2013a, Santarnecchi et al., 2013b, Santarnecchi et al., 2016) whereas bilateral prefrontal or sham tDCS, offline, even caused a detrimental effect (Sellers et al., 2015).

Creativity can be defined as “*the use of imagination or production of original ideas to create something (useful)*” (Barron, 1955, Sprugnoli et al., 2017). According to this definition, tDCS modulated creativity as measured with tasks assessing self-focused attention and mind wandering, inhibitory control, and divergent and convergent thinking, i.e., creative thinking (Lucchiari et al., 2018). This line of research suggests that stimulation may be “unleashing” creativity, mainly by modulating the bilateral temporal and prefrontal cortices. Placing the cathode over left and the anode over right cortical areas, in accordance with the hemispheric balance hypothesis, led to several studies finding positive results (Chrysikou et al., 2021, Hertenstein et al., 2019, Ivancovsky et al., 2019, Lifshitz-Ben-Basat and Mashal, 2021, Mayseless and Shamay-Tsoory, 2015). According to this hypothesis, a shift in interhemispheric balance resulting in a predominance of the right over the left hemisphere promotes creativity. However, recent neuroimaging and lesion studies suggest increased creativity occurs after protocols that aim to increase neuronal excitability in the left frontal areas associated mostly with verbal creativity, as measured with convergent and divergent thinking tasks, idea selection and reasoning (Cerruti and Schlaug, 2009, Green et al., 2017); notwithstanding, no changes in a verbal insight test after administering tDCS over the left and right anterior temporal lobe have been reported (Aihara et al., 2017). Enhanced creativity was also reported using bilateral prefrontal tRNS (Peña et al., 2019). Application of 10 Hz tACS over bilateral frontal areas was associated with improved figural creativity (Lustenberger et al., 2016) and marginally improved verbal creativity (Grabner et al., 2018), but no significant effects were found with 40 Hz tACS.

Pairing tES with cognitive and behavioral training regimens may be effective, while no consistent effect has been associated with stimulation alone (Cappelletti et al., 2013, Mancuso et al., 2016), or cognitive training alone (Martin et al., 2014). The approach aligns well with some of the presumed neural mechanisms of tES, which involve modulating ongoing neural activation patterns by strengthening or weakening these patterns via Hebbian synaptic mechanisms of neuroplasticity (Jackson et al., 2016, Kronberg et al., 2020, Reato et al., 2013), in which patterns of ongoing neural activity are selectively modulated and reinforced by stimulation (Gill et al., 2015). This approach has also been employed in clinical samples to target cognitive functions, like language processing (Nissim et al., 2020), motor learning (Wang et al., 2021), attention (Boroda et al., 2020), working memory (Jones et al., 2015), multitasking ability (Filmer et al., 2017, Manor et al., 2016), numeracy (Looi et al., 2017) and error awareness (Harty et al., 2014).

One obstacle has been the high degree of heterogeneity of the tDCS parameters, including stimulation polarity, intensity, and duration, electrode placement, and the temporal relationship of stimulation to behavioral training tasks (Ehrhardt et al., 2021, Martin et al., 2014, Weller et al., 2020). A second one is the identification of responders. Those whose performance was weaker at baseline may be more likely to experience performance gains than those whose baseline performance was stronger (Assecondi et al., 2021, Krebs et al., 2021, Sarkar et al., 2014, Turkeltaub et al., 2012). Finally, the degree to which training effects on a particular task during stimulation can generalize to related tasks is unclear (Andrews et al., 2011, Filmer et al., 2017, Looi et al., 2016).

## 7 Neuroenhancement and motor learning

With small magnitudes of effect, rTMS over the pre-supplementary motor area (preSMA) may enhance early motor learning processes, and over the supplementary motor area (SMA) may enhance the late motor learning process (execution speed-up) (Hean et al., 2020). Quadripulse stimulation (QPS) (Hamada et al., 2008, Hamada et al., 2009) was found to be one of the most effective methods, as it induces long-term effects in most studies with low inter-individual variability (Matsumoto and Ugawa, 2020, Tiksnadi et al., 2020). QPS may improve performance in a sequential learning task (Shimizu et al., 2020) as confirmed by human neuroimaging studies (Hikosaka et al., 1996, Sakai et al., 1998). However, due to its technical requirements, QPS will likely not be broadly applicable for neuroenhancement in healthy persons.

During tDCS over M1 implicit learning was accelerated compared with stimulation applied over prefrontal cortex or medial frontal cortex, or sham stimulation (Nitsche et al., 2003). However, as of 2022, very heterogeneous results emerge from the ∼500 publications on motor learning and tDCS, and no clear recommendations can be made here (Buch et al., 2017).

## 8 Neuroenhancement during sleep

Sleep is a particularly interesting target for NIBS and enhancement because stimulation takes place in a vital state for different brain and body processes (Grandner, 2017). One important function of sleep is memory consolidation, i.e., the reactivation and stabilization of fragile memory traces built during wakefulness (Rasch and Born, 2013). Accordingly, recall performance of declarative or non-declarative memories has been one of the most frequently employed paradigms to investigate whether modulating brain activity during sleep with tES elicits functional effects in healthy adults or those with a neurological disorder (Cellini and Mednick, 2019, Malkani and Zee, 2020). Brain oscillations that dominate non-rapid eye movement sleep (NREM) are thought to mediate the beneficial effect of sleep on various functions (Rasch and Born, 2013, Tononi and Cirelli, 2020). Non-invasive tES approaches that promote these oscillations have attracted tremendous interest in (i) establishing the functional role of sleep oscillations in brain and body processes, and (ii) restoring diminished functions in conditions that are associated with impaired sleep oscillations. In the laboratory environment, different tES applications ranging from oscillatory tDCS to sophisticated closed-loop tES systems that mimic the frequency of sleep oscillations have been mainly applied in healthy participants (e.g. Cellini et al., 2019, Jones et al., 2018, Ketz et al., 2018, Ladenbauer et al., 2017, Ladenbauer et al., 2021) but also in a few patient populations (Goder et al., 2013, Ladenbauer et al., 2017, Lafon et al., 2017, Prehn-Kristensen et al., 2014). Due to pronounced tES artifacts in the EEG, most laboratory studies analyze EEG data during intermittent, stimulation-free intervals and additionally employ behavioral outcome measurements to infer the effectiveness of tES for modulating sleep and sleep-related processes (Malkani and Zee, 2020).

Most previous studies have applied bilateral frontal, slow oscillatory tDCS (SO-tDCS) protocols, typically at ∼0.75 Hz mimicking the frequency of slow oscillations, to modulate sleep oscillations and sleep-related memory processes (reviewed by Grimaldi et al., 2020). For this protocol, two anodal electrodes are placed over the left and right frontal cortex (corresponding to the F3 and F4 locations) and the corresponding cathodes are placed over the left and right mastoid. The stimulation signal induces a current, which oscillates between zero and the maximal current intensity that is typically chosen to give maximum current densities of up to 0.522 mA/cm^2^. SO-tDCS is commonly applied for less than 30 min during the first period of stable NREM sleep, which is interleaved with stimulation-free, short intervals in order to analyze slow wave activity and sleep spindles. Less frequently used stimulation protocols employ (i) tACS around 1 Hz to entrain slow wave activity (Robinson et al., 2018) or tACS at 12 Hz to entrain sleep spindles (Lustenberger et al., 2016) and (ii) closed-loop setups whereby sleep oscillations are analyzed in real-time to trigger short bouts of SO-tDCS (Cellini et al., 2019) or 12-Hz tACS (Lustenberger et al., 2016).

The most consistent result across studies, which used SO-tDCS and tACS at slow wave frequencies, is increased slow wave activity in the stimulation-free windows compared with sham stimulation (e.g. Cellini et al., 2019, Jones et al., 2018, Ketz et al., 2018, Ladenbauer et al., 2017, Ladenbauer et al., 2021). However, some studies were unable to reproduce this effect despite a similar montage (e.g. Bueno-Lopez et al., 2019, Eggert et al., 2013, Koo et al., 2018, Sahlem et al., 2015). By contrast, effects on spindle activity seem variable, as well as the effects on declarative and non-declarative memory tasks (for a review see: Grimaldi et al., 2020). To date, stimulation parameters or inter-individual differences explaining the variability of effects on sleep and memory modulation have not been systematically investigated. However, it will be crucial to better understand these factors for a rational, individualized, and more successful design of sleep tES (Frohlich and Lustenberger, 2020, Koo et al., 2018).

There is an increasing interest in modulating sleep in unsupervised settings and ideally at home because (i) sleep quality might suffer through the laboratory environment, and (ii) laboratory settings are ill-suited for studying effects achievable via long-term neuromodulation during sleep. However, it is doubtful whether tES protocols as described above are suitable for neuromodulation outside of the laboratory. In particular, it is currently unclear whether a safe application can be enabled using tES considering that users are in an unconscious state and unintended side-effects might not directly be noted. Furthermore, tES during sleep is currently not available in an unsupervised setting because (i) it requires expert knowledge to correctly position the electrodes and reduce impedance, and (ii) precise stimulation requires an artifact-free EEG and individualized online monitoring to target the correct sleep stages and oscillatory processes. Because possible long-term adverse effects of nightly tES are currently not known, and the functional effectiveness of modulating sleep with tES seems to be limited as evaluated by behavioral markers of memory consolidation, tES-based approaches have gained virtually no traction for sleep applications outside of the academic setting. On the other hand, wearables such as Apple Watch, Samsung Watch etc. offer some surveillance including pulse rate and oxygen saturation with increasing accuracy. Along these lines, wearable EEG devices for home use have recently emerged that offer insight into brain activity and allow for possible closed loop applications (e.g. Ferster et al., 2019). These technologies provide an initial step towards future developments for tES home-use.

## 9 Transcranial electrical stimulation for well-being, reducing stress and burnout symptoms

Stress and burnout symptoms are indirectly connected with neuroenhancement. Some evidence exists that bilateral tDCS over the DLPFC is efficient in alleviating stress-induced creativity impairment (Wang et al., 2021). In this area, however, frequently popular information is mixed with science. Many companies marketing tDCS directly to use, indicate that devices are not sold for medical conditions but to enhance “wellness”, for example by decreasing general stress level, enhancing focus, or ameliorating burnout symptoms. When the psychology and behavioral sciences reporter, Miles O’Brien, underwent the procedure for a television interview, he said that his brain “seemed to turn on like a light bulb” and after, “*It was like a jolt of caffeine without the tense feeling*.” (https://www.pbs.org/newshour/show/gentle-electrical-jolt-can-focus-sluggish-mind). Given the strong placebo effects of NIBS, it is possible that sham stimulation would produce the same answer (see below in Section 11). At “Wellness Clinics”, business leaders and diplomats are invited to come and relax with healthy food, spa treatments and brain stimulation. Although no controlled scientific trials related to ‘improving well-being’ have been published, the framework of scientific tES data is used to claim that a decrease of subjective and objective stress levels and burnout improvement can be achieved that generally can improve the quality of life.

The scientific framework for tES does include improvements in properties related to well-being. For example, burnout, described as a 'state of vital exhaustion' was added to the 10th edition of the International Statistical Classification of Diseases and Related Health Problems (ICD-10: Z73.0:WHO, 2011). Left DLPFC anodal tDCS coupled with right orbital cathodal stimulation (F8) applied for three weeks improved burnout symptoms related to attention deficits (Van Noppen et al., 2020). Chronic stress, e.g. due to the pandemic (Castelo-Branco and Fregni, 2020) may benefit from tES, as well as some cases of post-COVID syndrome by tDCS at home stimulation (Eilam-Stock et al., 2021). Anodal and cathodal tDCS over the left and right DLPFC, respectively, for a single 30 min session in individuals with high but not with low anxiety profiles (Sarkar et al., 2014) improved performance in cognitive tests that could increase anxiety in participants with high anxiety profile, and was associated with lower cortisol levels than after sham tDCS. Three minutes of anodal and cathodal tDCS to the left DLPFC and right supraorbital area, respectively, significantly reduced ratings of unpleasantness in subjects exposed to distressing images, compared with sham stimulation (Boggio et al., 2009). Petrocchi et al. (2017) also observed an increase in positive affectivity in healthy subjects after 15 min of 2 mA tDCS over the left temporal lobe contrary to sham stimulation. Nevertheless, other studies found no mood-improving effects in healthy subjects (Morgan et al., 2014, Motohashi et al., 2013).

With regard to other possibilities, (Ironside et al. (2016) showed in healthy subjects that tDCS over the prefrontal cortex could reduce vigilance to threatening stimuli. Likewise, a similar treatment protocol improved frustration tolerance (Plewnia et al., 2015). Brunoni et al. (2013) found that a single three-minute session of 1.5 mA anodal / cathodal tDCS targeting the left / right DLPFC led to an increase in high frequency heart rate variability and a decrease in salivary cortisol levels that was not seen with sham or cathodal stimulation. Stimulation might have an effect on the autonomic nervous system and the hypothalamic–pituitaryadrenal (HPA) axis, however in a recent study, PAS and tACS did not elicit changes in saliva corticosteroid concentrations (Zulkifly et al., 2021b).

In summary, research data provided preliminary evidence for the value of anodal tDCS over the left DLPFC in the subjective and objective reduction of stress. Therefore, repeated application of tDCS might prove to be a useful and affordable therapy option in addition to conventional therapy, or in combination with other holistic methods (e.g. meditation) to help patients with burnout and chronic stress. However, further research is needed in randomized controlled trials to substantiate the preliminary evidence. Moreover, at the present state of tES science, due the limited scientific evidence, the application of brain stimulation for ‘wellness’ in healthy subjects is not recommended.

## 10 Sport, brain stimulation, doping

TDCS is seen as a potential ergogenic resource to improve muscular strength (Lattari et al., 2016, Lattari et al., 2020), and endurance (Okano et al., 2015, Lattari et al., 2018) in both nonathletes (Okano et al., 2015, Lattari et al., 2016, Lattari et al., 2020, Machado et al., 2019, Angius et al., 2018) and athletes (Sales et al., 2016, Hazime et al., 2017, Vargas et al., 2018).

With regard to sport and NIBS, the most frequently targeted area is the left DLPFC, because it is responsible for the inhibition of motor areas. Generally, this area has a role in the modulation of plenty of functions and processes, such as emotion, mood, and memory. Therefore, it is difficult to measure the impact of a given stimulation on just one parameter. The left DLPFC is an area that acts in controlling fatigue and exertion, thus, it is one of the most important areas involved in deciding whether or not to continue exercising (Lattari et al., 2016, Tanaka et al., 2013). Physical fatigue is a complex phenomenon, and factors such as perceived effort and central inhibition may be involved. In fatigue, there is an increase in brain activity (beta power, measured by EEG) and a greater synchronization between the left and right DLPFC (Tanaka et al., 2013). Fatigue may be compensated in the DLPFC when there is a decrease in motor cortex activation due to the presence of central fatigue induced by exercise (Menotti et al., 2014). In line with this, Lattari et al. (2020) found that anodal tDCS applied to left DLPFC and cathode over the Fp2 at 2 mA for 20 min improved volume load and perceived exertion compared with sham tDCS and with tDCS using a reversed montage for lower limbs exercise in healthy young individuals. Twenty minutes of anodal tDCS at 2 mA applied over the left DLPFC (cathode over the Fp2) increased the tolerance to the exercise performed in the cycloergometer with maximum load in moderately active women compared with sham stimulation (Lattari et al., 2018). This finding implies that the modification of the neuronal activity in the left DLPFC could enhance endurance exercise performance by maintaining the volitional impulse to the motor cortex. However, other studies have failed to show significant tDCS effects on exercise performance (Barwood et al., 2016, Kan et al., 2013, Muthalib et al., 2013, Angius et al., 2015). Early reports on facilitation of motor learning (Nitsche et al., 2003) were recently confirmed and extended to improvement of jump performance (Grosprêtre et al., 2021). If data on skilled musicians could be transferred to sports, a generalized conclusion could be that the chance of improving gross motor power or endurance might be higher as compared to skilled sports requirements (Furuya et al., 2014). Furthermore, because the majority of tDCS studies on this field used a single stimulation session, studies using repeated sessions are warranted.

Using tDCS to improve performance in sport/exercise could be considered a form of “neurodoping” (Davis, 2013, Banissy and Muggleton, 2013, Colzato et al., 2017, Park, 2017), as it associated with physical risks, behavioral and ethical issues. Nevertheless, tDCS has escaped so far the standards of the world anti-doping agency (WADA), which are entirely focused on drugs: https://www.wada-ama.org/sites/default/files/resources/files/2021list_en.pdf. Since there is reasonable evidence that some tES protocols may improve motor performance in normal persons, it may thus be seen as a ‘doping method’, without legal regulations so far. Moreover, so far, there is no “biomarker” for detecting its use. It is suggested that tDCS should not be prohibited but WADA should adopt a similar approach to monitor its use as it currently does to caffeine (Pugh and Pugh, 2021). Prohibition would encourage the use of unsafe devices and protocols or stimulation. As part of that monitoring, WADA could work with stimulation companies developing tDCS devices to ensure that athletes are properly informed about the applications, risks and effects of tDCS.

## 11 Neuroenhancement and the placebo effect

Developing more effective sham tES procedures is crucial for assessing neuroenhancement by tES (Davis, 2014, Fonteneau et al., 2019, Greinacher et al., 2019, O’Connell et al., 2012, Turi et al., 2019). Although most tES studies use sham tES protocols, few have directly investigated placebo or nocebo effects (e.g., BinDawood et al., 2020, Petersen and Puthusserypady, 2019, Turi et al., 2017, Turi et al., 2018). Similar to other placebo interventions (Colagiuri et al., 2011), sham tES protocols may induce complex psychobiological responses. In Parkinson patients expectation has shown to be able to provide a dopamine release in the striatum (de la Fuente-Fernandez et al., 2001).

A placebo effect can be defined as an enhancement and a nocebo effect as an impairment of a given outcome variable during and/or after the application of sham tES. The outcome variable can refer to any biomarker and may extend to cognitive functions or (indirect) physiological responses (Petersen and Puthusserypady, 2019, Turi et al., 2018). Specific dopamine and opioid circuits respond to the expectation of benefit during placebo drug administration, inducing measurable physiological changes (Zubieta and Stohler, 2009).

Both conscious or unconscious factors can contribute to the generation of placebo effects (Wager and Atlas, 2015). To begin with, most participants experience a moderate amount of cutaneous sensation (e.g., itching, tingling) with conventional tES, which is supposed to be mirrored during sham tES (Fertonani et al., 2014, Matsumoto and Ugawa, 2017). For tACS, in addition to the cutaneous effects, visual flickering effects (i.e., phosphenes) may occur (Lorenz et al., 2019, Schutter and Hortensius, 2010, Turi et al., 2013). Thus, blinding as part of the experimental set-up may be ineffective in tACS protocols with frequencies between ca. 5 and 40 Hz if no attention is paid to phosphenes (Turi et al., 2013).

A commonly used sham tES method is the ‘fade-in, short-stimulation, fade-out’ (FSF) protocol (Ambrus et al., 2012), though it is important to note some forms of ‘sham’ that include ongoing low intensity, microampere stimulation have been shown to have biologically active effects (Nikolin et al., 2018). Early tDCS studies suggested that participants were unable to distinguish between the real and FS or FSF protocols (Gandiga et al., 2006). However, subsequent studies provided accumulated evidence that the FSF protocols cannot completely blind the commonly used real tDCS protocols (Greinacher et al., 2019, O’Connell et al., 2012, Turi et al., 2019), in particular when intensities higher than 2 mA and stimulation durations longer than ten minutes are used.

Subjectively experiencing the tES-induced perceptual adverse effects can contribute to the generation of placebo or nocebo effects. Besides, a general limitation of single-blind studies is that they may inadvertently influence the interaction between the operator and the participant (O’Connell et al., 2012, Palm et al., 2013). For example, the operator might unintentionally give different instructions during the real than during the sham sessions. Instructional manipulations combined with sham tES could induce cognitive placebo or even nocebo effects in healthy individuals (Turi et al., 2018). Ineffective blinding may not only interfere with attentional processes during tES, but might lead to psychobiological responses through expectations (Colagiuri et al., 2011, Oken et al., 2008, Braga et al., 2021). Experimentally manipulating the expectancy of tES outcome, e.g., by placebo-inducing written instructions combined with sham tES can on itself influence cognitive functions in young healthy adults (Turi et al., 2017, Turi et al., 2018). The combination of sham tES with conditioning (i.e., surreptitiously manipulating the trial-by-trial feedback of the cognitive task) led to a placebo effect both in the subjectively reported cognitive effects, as well as in the instrumental learning performance (Turi et al., 2018). Placebo effects might be highest in children and lowest in patients with dementia (Benedetti et al., 2011, Gniss et al., 2020). Because a substantial amount of the sham tES protocols still do not provide complete blinding, using other control conditions, such as stimulation of ‘irrelevant’ brain areas or using other types of stimulation, are also suggested.

## 12 Neuroenhancement in paediatric population

Several randomized, placebo-controlled trials suggest beneficial effects of tDCS over cognitive functions in the treatment of dystonia, refractory epilepsy, attention deficit/hyperactivity disorder, and autism in children/adolescents (for reviews see: Andrade et al., 2014, Doruk Camsari et al., 2018: Finisguerra et al., 2019, Lee et al., 2019, Muszkat et al., 2016, Palm et al., 2016, Rajapakse and Kirton, 2013, Rivera-Urbina et al., 2017, Salehinejad et al., 2019). Children and adolescents differ from adults in the conductivity of their skull tissues, in white and gray matter content, and in CSF volume, and when compared to adults they also have a smaller brain-scalp distance, all of which influence the distribution and intensity of the electric field. For example, reducing the HD-tDCS 4x1 ring circumference from 5 cm to 2.5 cm for use on children’s heads reduced peak electric field values to intensities broadly more comparable to 4x1 HD-tDCS with a 5 cm ring in adults (Kessler et al., 2013). The age-related anatomical and physiological changes of the brain may even reverse stimulation effects (Moliadze et al., 2015b, Moliadze et al., 2018) at about half of the intensity which is known to reverse effects in adults (Batsikadze et al., 2013). Recent results support the notion of tDCS dosage administration based on subject-specific models (skull thickness, tissue electrical conductivities) in children (Hunold et al., 2021).

Studies conducted in children have not reported more adverse effects than observed in adults (Andrade et al., 2014, Moliadze et al., 2015a, Moliadze et al., 2015b, Splittgerber et al., 2020a, Splittgerber et al., 2021, Splittgerber et al., 2020b). Special attention should be paid to muscle jerks on awakening by questioning adolescents and their parents in order to detect evidence of myoclonic epilepsy, which may indicate greater risks with tDCS (Sierawska et al., 2020, Splittgerber et al., 2020b). In terms of tolerability, there are no special concerns for using tES in pediatric patients. In terms of safety, limited data exists concerning long-term after-effects of tES in children/adolescents.

In summary, the risk in using tES for neuroenhancement in a pediatric population, especially as home-stimulation, has not been sufficiently studied. “*Children [cannot] be considered as ‘small adults’ when testing medical interventions*” (Davis, 2014), see also (Maslen et al., 2014). Therefore, the findings from studies in adults cannot simply be extended to studies in children since the evidence regarding adverse effects in pediatric patients is still limited. Researchers have to take into account that because of the neuroanatomical and physiological differences between the brains of children and adults, the tES parameters applied in adults will have a potentially greater impact when applied to the pediatric brain. Monitoring the occurrence of potential cumulative effects and possible impairments in the paediatric population is necessary. At present, tES in children for the purpose of neuroenhancement is discouraged.

## 13 Neuroenhancement or treatment? Neuroenhancement in the elderly and cognitively impaired population

At the other end of the age spectrum, a growing elderly population has increased the prevalence of several neurodegenerative disorders like dementia due to Alzheimer’s (AD) and mild cognitive impairment (MCI). “Normal” aging itself is associated with progressive decline in cognitive functions like memory, learning, and executive function (Prehn and Flöel, 2015). The direct and indirect costs due to “normal” cognitive deterioration not only cause a profound loss of quality of life for the individuals, but present significant challenges to the sustainability of the welfare system, an issue exacerbated in neurodegenerative disease (Colzato et al., 2020).

Given the dearth of pharmacological interventions for cognitive enhancement in the elderly population, non-pharmacological avenues include cognitive training, physical activity, nutraceuticals, and NIBS techniques, particularly tDCS and rTMS (Chu et al., 2021), have attracted increasing attention (Prehn and Flöel, 2015). Anodal tDCS tailored cognitive training with stimulation of the left DLPFC may be beneficial for several subdomains of cognitive dysfunction in patients with MCI or AD (Chu et al., 2021) in line with cognitive training with anodal stimulation of the right temporoparietal cortex, which may have positive effects on object location memory in patients with MCI (De Sousa et al., 2020).

The variability of NIBS effects in older adults may be greater due to aging-related brain atrophy. Furthermore, the increased intracranial CSF content leads to a broader current distribution. However, using predictive current flow models based on structural brain images may help personalize the electrode positions with resulting improvement in target localization (Antonenko et al., 2021).

## 14 Personalized neuroenhancement using cognitive and behavioral fingerprints: Opportunities and challenges

At present, cost and time requirements will prevent a general, broad application of neuroenhancement using NIBS techniques, mainly the application of rTMS. Intuitively, the general approach to precision medicine instead of a one‐dose‐fits‐all mode can be translated to neuroenhancement applications in both healthy individuals and patients, and even more so when utilizing NIBS techniques and technology where individual differences in the response to brain stimulation protocols constitute the norm rather than the exception (Bikson et al., 2018, Corp et al., 2020, Santarnecchi et al., 2016). In general, however, individualization in the context of precision medicine needs at least an individual MRI and current flow calculations, e.g. using programs like SimNibs. Such “fingerprinting” of individuals, also on the basis of resting-state neuroimaging (e.g., MRI, fMRI, DTI) and electrophysiological (e.g., EEG) data is becoming a standard in neuroscience, with evidence of the possibility to accurately identify unique individual features of brain structure and function that are also of particular relevance for explaining interindividual variability in cognitive performance (Finn et al., 2015, Ozdemir et al., 2021). Individualized neuroenhancement solutions should start with selecting the target. For example, in a memory enhancement intervention the most appropriate target region/network for the participant would be selected depending on his or her imaging/electrophysiology correlates of memory performance, and individualization of stimulation intensity based on parameters such as cortical excitability, biophysical modeling and other features obtained, e.g. by combined TMS-EEG recordings of TMS evoked potential amplitudes for non-motor regions. Recent retrospective studies have shown the importance of proper selection of individual rTMS targets within the left DLPFC on the basis of fMRI functional connectivity patterns in patients with major depression (Siddiqi et al., 2021). Evidence is also available for genetic factors that predict the response to stimulation, such as BDNF polymorphisms (Jannati et al., 2017), as well as for baseline cognitive performance (Krause and Cohen, 2014, Kadosh, 2014). Efforts have also been made to prospectively tailor stimulation parameters on the basis of individual brain or cognitive features, for instance by personalizing the stimulation frequency of tACS by targeting the individual alpha frequency (Kasten et al., 2016), tailoring stimulation frequency and intensity of tACS based on cognitive ability (van Bueren et al., 2021) or by identifying optimal TMS targets via analysis of individual connectome data. Overall, better results with personalized neuroenhancement approaches seem to be widely accepted (Horn and Fox, 2020), and the required technology is partially ready to be included, e.g., portable EEG headsets that will enable remote brain activity recording and treatment adjustment, wearables to track physiological parameters (e.g., heart rate, sleep patterns), as well as analytical methods for the creation of individual target maps accounting for differences in anatomy and function (Kasten et al., 2019, Ruffini et al., 2020).

However, questions remain. One aspect to consider is the temporal framework of personalization approaches and the issue of *state-* vs *trait-based* personalization. While genetic features constitute a trait since they are unlikely to change, changes in functional brain features, such as EEG activity and fMRI connectivity, are to be expected over the course of weeks, days, hours and even shorter intervals. Optimizing for individual features known to be fluctuating over time might not necessarily lead to an increase in effectiveness but actually introduce more variable effects between or within sessions, unless repeated baseline assessments are performed, or real-time time manipulation of brain stimulation parameters is achieved via simultaneous brain activity recording (e.g., closed-loop EEG-tES or EEG-TMS; Zrenner et al., 2020). Related to this issue is the aspect of the generalizability of effects in larger populations. This is particularly relevant when promoting novel therapeutic solutions. Paradoxically, the adoption of a normative, population-level template describing, for instance, the average patterns of activity during a memory task recorded in a large sample of patients with mild-to-moderate AD, might constitute the optimal balance between personalization and generalizability, by increasing the chance that patients with similar conditions will benefit from the proposed stimulation approach even though it might be slightly sub-optimal at the individual level. Finally, barriers to the adoption of technology and the approval of novel clinical interventions should also be considered, including costs and burden of exposing patients to additional data acquisition sessions (e.g., fMRI, PET, TMS-EEG). In summary, neuroenhancement will benefit greatly from a better understanding of individual characteristics and from the development of the necessary technology to increase temporal and spatial resolution of individual “fingerprints”. A careful cost-benefit analysis is also necessary to prevent unnecessary complexity that might prevent large-scale adoption of neuroenhancement interventions.

## 15 The media hype about neuroenhancement and brain stimulation

The phenomenon of hyperbolic media reports on neurotechnology or neuroscience in general is not new (see e.g., Racine et al., 2017). However, the proliferation of such reports and the explicit link of neurotechnology with prospects of enhancement, whether in the form of military funding for research that might provide an edge, or in terms of futuristic visions of a “posthuman” society, can generate a backlash. Namely, an overarchingly positive (“Hype and Hope”) attitude towards enhancement was met head on by an equally broad negative (“Doom and Gloom”) attitude (see Voarino et al., 2016). However, public interests and democratic deliberation are best served if new neurostimulation technologies are viewed not through any one all-inclusive lens but on a case-by-case basis, so that self-regulation of the academic community and industry standards can be accomplished in benign cases, whereas for more serious cases, regulation could be approached by timely “agenda setting.” Polls conducted by social scientists have tracked what the general public considers to be “most important problems” for society, and research has shown that such “important problems” tend to closely follow media coverage (Birkland, 2005).

Early media discussions about neurostimulation have been optimistic, with little explicit discussion concerning ethical issues, therapeutic limitations, or adverse effects that could result from their wide-spread use (Dubljevic et al., 2014, Racine et al., 2007). Racine and colleagues analyzed print media coverage on neurostimulation between 1994 and 2004, and reported a distorted depiction, which often included first person narratives of patients, and sometimes celebrities, resembling “miracle stories.” The media used buzzwords or short catchy phrases, such as “currents of hope” and “magnetic appeal,” to convey the message that neurostimulation is a “scientific breakthrough.” The early reporting was fairly general, with only 20% (*N* = 46) of the sample in Racine and colleagues’ study specifically referring to a NIBS technique such as TMS.

Further empirical work on media representation of brain stimulation was influenced by the appearance of commercial tDCS devices on the market, and by the specific use of enhancement claims in order to avoid FDA regulation of therapeutic devices. In 2014, Dubljevic and colleagues reported strong and potentially misleading statements about the real-world effects and applicability of tDCS, even in otherwise serious news outlets (e.g., “*schoolchildren who struggle to grasp mathematics could benefit from having their brains roused with electricity*” [*The Guardian*, April 11, 2010]).

The concept of agenda-setting by the media (McCombs and Shaw, 1972) describes how media coverage impacts what the general public considers to be a major issue. Bernard Cohen (Cohen, 1963) offered what is considered to be the classic summary of agenda setting: “*The press may not be successful much of the time in telling people what to think, but it is stunningly successful in telling its readers what to think about*” (p. 13). Issues that are most salient – defined by frequency, location, and/or length of coverage – in the media become part of the public discourse (Birkland, 2016). The more coverage an issue receives, the more the public pays attention (McCombs et al., 2014). Media are further found to impact the public’s awareness of an issue based on *how* they report on a subject (McCombs and Shaw, 1972).

While neurostimulation techniques could be therapeutically legitimate for a number of people with diverse health concerns, access to care may be influenced by misleading media portrayals that offer overly positive or overly negative perspectives. Positively biased information may lead to unrealistic expectations, which fail to materialize for the majority of people, ultimately leading individuals to see their results as a “let-down.” For example, media that paint a brain stimulation technique such as rTMS as “life-changing” or a “miracle cure” for depression may unwittingly set up those people who were treated and experienced only mild benefit, for disappointment (Dubljević et al., 2020). Similarly, as in situations of direct-to-consumer advertising, such stories could encourage patients to seek out treatment, in an era of self-diagnosis, that is not appropriate for them. On the other hand, negative perspectives may discourage patients from seeking or continuing treatment, even if the technology has legitimate, potential benefits. For example, overly negative testimonials or op-ed pieces from people who were disillusioned with NIBS might discourage others from seeking this treatment, even in cases where they could be suitable candidates. Given the implications of media representation for potential patients, media should take a balanced approach to discussing health technologies such as NIBS.

## 16 NIBS in military environments for performance enhancement

Military organizations have been interested in NIBS for some time for its possible therapeutic use in treating diseases and injuries typical of military occupations (Oberman et al., 2020), but they have also recognized the usefulness, even the need, to explore cognitive enhancement for national security (Malish, 2017). It has been pointed out that with advancing technology, the modern soldier requires more complex mental skills than in the past, but that cognitive performance degrades in combat situations, and that NIBS may provide the means to counteract that degradation (Davis and Smith, 2019).

This interest was present early on: for instance, in 2002, the US Defense Advanced Research Projects Agency (DARPA) invested in using TMS for performance enhancement at a time when only a few TMS studies had even reported such effects, calling them “paradoxical,” as they were unexpected. As part of a larger effort to remediate the cognitive effects of sleep deprivation, a common problem in military situations, DARPA had funded a few groups to make the attempt using TMS. One group from Columbia University succeeded, by focusing specifically on working memory, a process central to executive cognitive function, in which deficits caused by sleep deprivation typically manifest as slowing of response and lapsing (missed trials) during the task. This was achieved in a succession of steps, beginning with a study investigating the experimental parameters that might lead to performance enhancement rather than disruption (Luber et al., 2007). Targeting nodes of the fronto-parietal executive network (not called that at the time) based on group fMRI activations of a delayed-match-to-sample (DMS) task, trains of rTMS to the parietal cortex during the delay period of the task at 5 Hz (but not 1 or 20 Hz) resulted in speeded performance compared with sham. This effect was reproduced in a different group of subjects again with stimulation during the delay phase of the task, but not during the test phase. In the next step, using an fMRI network for neuronavigated targeting that was activated by the DMS task but decreasing in its activation with sleep deprivation, an acute remediation of sleep deprivation-induced response slowing was seen in sleep deprived subjects for 5 Hz rTMS applied within the fMRI network, but not outside (Luber et al., 2008). In a third step, this effect was prolonged well past the application of rTMS. Here, rTMS was applied in a number of sessions over the course of the two-day sleep deprivation period while the DMS task was performed. A day after the last rTMS was given, a group of sleep deprived subjects who had received sham stimulation showed the typical slowing and lapsing deficits, while a group who had received active rTMS performed deficit-free (Luber et al., 2013). Overall, this was an early and concerted effort by a military research organization to develop NIBS applications useful in a military environment: a paradigm was developed, using fMRI-guidance, co-activation of targeted cortex using concurrent task performance and TMS, and multiple sessions to create a cumulative effect, which resulted in a long-lasting TMS-caused cognitive enhancement. While DARPA has gone on to study cognitive enhancement using a more invasive approach (e.g., DBS to affect memory: Deadwyler et al., 2017), other institutions such as the US National Institute on Aging adopted the NIBS paradigm to explore enhancement of working memory in aging populations (e.g., Beynel et al., 2019).

Military organizations have also investigated the use of tES for performance enhancement. For example, the U.S. Air Force Research Laboratory created a program to actively seek enhancement technologies to help bridge the gap between the ever-stronger computational capacities of machine interfaces and the limitations of their human operators (McKinley et al., 2012). For example, they sponsored a tDCS study while subjects were being trained to search complex simulated radar images of terrain containing buildings and vehicles for military targets and increased their accuracy by about 25% over non-stimulation conditions, suggesting the potential usefulness of tDCS in accelerating training (McKinley et al., 2013). Another study showed the efficacy of tDCS to train personnel in threat detection using virtual reality software, where tDCS increased perceptual sensitivity in detecting threats significantly compared with non-stimulation conditions (Parasuraman and Galster, 2013). A recent review by an Australian military research organization aggregated tDCS enhancement studies by typical psychological categories such as attention, perception, memory, and reasoning, and also by communication skills, physical performance, and resilience, and discusses their potential benefits in the military (Davis and Smith, 2019). The review also addresses possible risks, and offers a number of ways to move forward, although another recent review cautions that at the current state of tES research, while promising for military use, is far from ready in an operational environment (Feltman et al., 2020). Also important in this regard are ethical concerns about coercion and autonomy, and risks to others in battlefield conditions. These concerns and related questions have also been discussed in previous reviews (Sehm and Ragert, 2013, Levasseur-Moreau et al., 2013).

## 17 Neuroenhancement and self-applied stimulation: More risks outside the lab environment? Neuroethics and neuroenhancement

The non-professional or “lay” use of electrical stimulation has a long history dating back several centuries in both Europe and the United States (Kadosh, 2014). In the late nineteenth and early twentieth century, handheld devices known as “medical batteries” that provided either alternating or direct current were sold to both physicians and the public with claims of treating a wide variety of ailments and disorders (Peña, 2003, Currier, 2004, Waits, 2013, Wexler, 2017b). Aside from the marketing of a handful of products in the 1920 s and 1930 s for “rejuvenation” and “reinvigoration,” the notion of the use of electricity for enhancement—cognitive or otherwise—appears to have been largely absent from most historical marketing claims (Wexler, 2017a).

The contemporary movement regarding the lay use of tES for enhancement began in 2011, when lay individuals began to construct tDCS devices in their homes. Since then, dozens of companies, largely based in the U.S., have marketed ready-to-wear tES devices for brain optimization and cognitive enhancement (Wexler, 2015, McCall et al., 2019). To date, three empirical studies have been conducted to better understand users of self-directed home tES devices (Jwa, 2015, Wexler, 2016, Wexler, 2018; for review see Wexler, 2020). All found that most users were male and largely based in North America. Two earlier studies found that the typical user was in her/his 20 s or 30 s, whereas the more recent study (Wexler, 2018) reported a mean age of 45. While some individuals used stimulation at home for treatment purposes (most commonly depression), most subjects reported using tES for cognitive enhancement, specifically for improving focus and memory. Notably, however, those who have utilized tES for enhancement do not report it to be particularly successful (Wexler, 2018).

Further research is needed on the potential of tES to produce neuroenhancement in otherwise healthy individuals (normally functioning brains) and outside of controlled laboratory environments, as well as on the potential for adverse events heretofore not identified in human trials and to clarify if and when benefits in one cognitive domain occur at the expense of other cognitive functions (Iuculano and Cohen Kadosh, 2013). Such considerations may be device or protocol specific. For example, one double-blind, sham-controlled study suggested that using a non-CE-marked commercial tDCS headset in healthy young adults produced detrimental effects on cognitive abilities instead of the promised ones, i.e., “*let the force of electricity excite your neurons into firing faster*” (Steenbergen et al., 2016).

The most likely adverse effect that may occur to inexperienced users is a skin irritation due to improper electrode impedance, missing electrolyte, tap water used as electrolyte, dried out sponge electrodes or application/removal of electrodes while the device is switched on (Antal et al., 2017, Woods et al., 2016). Even those lay users of tES devices, which do adequately provide dose control (setting restrictions on their use), may fail to position the electrodes over the correct cortical target due to lack of anatomical knowledge, or may apply the wrong dosage with respect to tES intensity and duration, or may reverse stimulation polarity by switching anode and cathode, causing unintended effects (Fitz and Reiner, 2015) such as decrease performance in an intelligence test (Sellers et al., 2015). Furthermore, the time spent at home by an individual under stimulation may not be limited by some devices (in contrast to remote-supervised use that provides for remote control; Charvet et al., 2020). This results in unknowns with regard to outcomes, in the attempt to “overclock” one’s own cognitive abilities, for example for improving online gaming performance (Santarnecchi et al., 2013a, Santarnecchi et al., 2013b). Here, non-linearities of effects due to extended dosages, including stimulation intensity, duration, and inappropriate intervals in case of repeated stimulation might play a role (Agboada et al., 2019, Agboada et al., 2020, Batsikadze et al., 2013, Monte-Silva et al., 2013, Mosayebi-Samani et al., 2019, Mosayebi-Samani et al., 2020). While there is no evidence tES or TMS can be addictive (and indeed some research of using tES / TMS to treat addictions), the potential for individuals (otherwise prone to addictive behavior) to excessive auto-stimulation should be studied (Santarnecchi et al., 2013a, Santarnecchi et al., 2013b).

Many scholarly and media articles have portrayed the home use of tES as increasing, although an empirical assessment of the phenomenon in “real-world” scenarios is challenging. It is clear, however, that the home use of tES has not become mainstream at this time, but rather has remained limited to small groups of users. While devices continue to be sold—one online survey of tES devices claims to have sold tens of thousands of products per year to consumers (Waltz, 2019)—the effectiveness of these devices, and the value they provide to consumers, remains an open question. Still, companies continue to bring new devices to market, in many regions aided by the lack of strict oversight from regulatory bodies regarding products marketed for enhancement or wellness purposes.

## 18 Neuroenhancement and regulating the marketing of devices

Most nations clearly differentiate the regulation of medical devices from that of other instruments and appliances. This distinction stems from the high standards for regulating the marketing of devices for medical diagnosis and treatment. While the regulation of medical devices is often determined by their perceived risk level, whether a device is considered to fall under medical device regulations (MDR) is not governed by risk, but rather by its ability to diagnose or treat a medical condition (e.g., a chainsaw would not be regulated as a medical device despite posing a clear potential health risk). Therefore, the perceived risk of a device cannot be the basis to decide if it should be regulated as a medical device. Rather, long-standing and well-developed regulations provide guidance on this topic.

Neuromodulation devices marketed for wellness and cognitive enhancement without explicit connection to a disease are not considered medical devices in all well-known U.S. jurisdictions. However, in some cases (notably the updated EU MDR) “*products without an intended medical purpose*” may fall under medical device regulations; in addition practices developed to ensure medical device quality (e.g., risk management) may be adopted through voluntary industry standards (Bikson et al., 2018). In the EU all tES and TMS devices have been treated as Medical Devices since May, 26th 2021; this includes any electrical, magnetic or electro-magnetic stimulation device for cognitive enhancements. The term *Medical Device Directive* (MDD) was taken out of action (beside the transition period of existing Medical Devices) by May, 25th 2021. According to the new MDR, tES and TMS devices will be regulated as stated in MDR ANNEX XVI (6) and at the official pages of the EU https://ec.europa.eu/health/md newregulations/getting ready/manufacturers devices without intended medical en. However, the Common Specifications are not published yet. Those will give a clear understanding how those devices should be verified and tested with respect to risks and safety.

When specific agencies are assigned to regulate medical devices (e.g., the FDA in the USA) they: (1) specifically regulate manufacturers and not the practice of medicine (e.g., physicians) or consumer behavior, and (2) focus on regulating the claims made by the manufacturer (also called the label) regarding specific medical uses. Medical devices are also regulated in how they can be sold, for example only to medical centers, to individuals under a prescription, or over-the-counter (OTC). There are currently hundreds of OTC products available, and in many cases, the output of these OTC products exceed the output capacity of low intensity tES devices (Bikson et al., 2018). In the context of MDR, it is critical how instruments are accessed and that a well-established framework exists for this purpose (e.g., human factors testing). For non-medical devices, such restrictions are largely absent with few exceptions, usually when there is an established and exceptionally high risk of harm (e.g., firearms, vehicles, illegal drugs). However, just a potential for harm is not enough basis for regulation (e.g., hammer and nails). Even in the few cases where special regulations exist for consumer devices, it is rarely under (subverting) MDRs. It would be an unheard-of situation, when physicians would be required to prescribe (or otherwise limit access to) a device that is not regulated as a medical device. The EU’s updated MDRs take an explicit and special stance on “*Equipment intended for brain stimulation that apply electrical currents or magnetic or electromagnetic fields that penetrate the cranium to modify neuronal activity in the brain*”, regulating such devices under MDR. Those devices are defined as Medical Devices without an Indented Use (Medical Claim).

As noted, governments (mainly outside the EU) only rarely restrict sales of non-medical devices to consumers, except for firearms and such. The theoretical potential for a non-medical device to be used by an individual for self-directed medical care has no bearing on how the device is regulated. For neuromodulation devices there may be a potential for both medical (e.g., insomnia) and wellness (e.g., “good night sleep”) uses. This cannot in-itself justify regulation for the latter if there are no further, specific reasons (although we acknowledge that selling such devices without any evidence for the efficacy of the particular device could serve as a reason). This raises the conundrum that a device, such as a tDCS device marketed for wellness (thus not as a medical device) may be obtained by an individual and used for self-directed treatment.

It is therefore not correct to insinuate that there is no legal guidance for consumer neuromodulation, at least in most of the countries. First, as noted above there is an extensive framework for distinguishing between medical devices and wellness devices. Indeed, in some cases the medical device regulator even provides an explicit mechanism to confirm that a device is not a medical device, and is thus not regulated as such (e.g., the 513 g mechanism for the US FDA). Second, non-medical devices are subject to a range of regulations including those dealing with fair marketing (e.g., the Federal Communications Commission - FCC in the US). Third, in some regions additional regulations are applied to non-medical neuromodulation devices (e.g., in the EU).

While we think that companies that sell brain stimulation devices to the public should be based on scientific evidence to support their own product. We acknowledge that any discussion on increasing the burden of regulation on companies or individual users must be conducted with regard to the spectrum of existing regulations and the rationale for both imposing and limiting regulatory burdens.

## 19 Conclusions and recommendations

While substantial research on low intensity tES effects on human learning and memory has already been conducted, recent reviews have noted methodological variability, cases of lack of rigorous experimental control, and variable outcomes within and between studies. Advancing the science of tES will benefit from systematic and coordinated evaluation of a huge parameter space (type of stimulation, montage, dose of stimulation, number of repetition and breaks between them, and for the case of tACS, frequency – or the combination of frequencies – and phase), further clarification of the underlying neural mechanisms, and possibly individual adaptation of interventions based on this knowledge. This is closely related to the need to develop optimized sham protocols, e.g., based on electric field simulation (Neri et al., 2020) and appropriate evaluation methods (Fonteneau et al., 2019, Turner et al., 2021) to assure appropriate double blinding in forthcoming research and clinical studies. Nonetheless, these studies have shown that there are potential positive effects on behavior and brain function, but the current “state-of-the-art“ leaves open questions about translational application. For example, more stimulation (e.g., stimulation with higher intensity and for a longer time) might not be better (Gamboa et al., 2010) and can even reverse the desired effects. Short-term as well as long-term rebound effects could be at play: homeostatic processes are an integral part of the brain’s function.

Furthermore, the translation of laboratory findings of selected aspects of cognitive or behavioral enhancement by NIBS to real-life contexts is largely unexplored. For instance, we might observe improved performance in a working memory task after applying a form of NIBS in the laboratory, but there are no data if this would translate to improved performance at work or school when applied in settings outside the laboratory. Additional research on the effects of NIBS is needed, including how use and outcomes may vary in “real world” settings. Effects observed using restricted (and abstracted) laboratory tests may not generalize. Further research should also consider the long-term effects of repeated NIBS sessions.

Reports in social media that promise dramatic improvements in cognitive functions that do not accurately reflect scientific findings, or do not indicate any identified risks, may unduly influence healthcare providers, patients, and families when weighing the harm-benefit ratio. This includes reporting when a given outcome is anecdotal or from a non-controlled trial (given placebo and nocebo effects).

To maximize reliability, tES devices marketed for cognitive enhancement should be engineered and manufactured to standards adopting appropriate practices established for medical devices, whether guided by industry standards (Bikson et al., 2018) or national regulators (as the new law in the EU). There will be Common Specifications as mentioned in EU’s rolling plan item #3 in https://ec.europa.eu/health/sites/default/files/md sector/docs/md rolling-plan en.pdf by next year. According to this, manufacturers have to adapt to these regulations within 6 months after publication. This will include tES devices for cognitive enhancement. Notwithstanding the tolerability and safety of tES as used in human trials (Antal et al., 2017, Bikson et al., 2018), efforts to provide tES directly to consumers (outside of remote-supervised use; Charvet et al., 2015) must consider the potential for improper use (Wurzman et al., 2016) which in turn can produce unpredictable outcomes.

The consensus among experts is that low-intensity tES is safe so long as accepted protocols (dose, inclusion/exclusion, proper electrode attachment) are followed. Devices that do not respect prior limitations on these factors, cannot claim to be “safe”. Based on our previous recommendations (original document:

https://wfneurology.org/news_events/archived-news/2016–01-25-ifcn), if low intensity tES is considered for neuroenhancement, the following conditions have to be taken into account:

- We recommend that tES in the treatment of a medical indication (at home or in the clinic) be done with a medical grade device and under on-site or remote supervision (Charvet et al., 2015) of a medical provider or trained personnel. The self-directed use of tES for medical indication – namely without a prescription or remote-supervision – requires device specific consideration of human factors and associated specific federal regulation. For example, in the United States, such devices are approved as Over-The-Counter (OTC).
- In medical applications, a necessary and sufficient condition is the use of protocols, which have been demonstrated in peer-reviewed clinical trials to be both safe and efficacious. The manufacturer should provide this information in the user manual, based on clinical evaluations. If a manufacturer makes any device claims that would be considered by relevant medical-device regulatory agencies as medical claims such that the device is a medical device, then evidently all standards for medical devices set by the regulatory agency will apply. In the case of non-medical claims (e.g., wellness or enhancement claims), regulation by medical-device agencies will vary. Manufacturers must follow ethical and best practices in marketing devices, including not misleading users by referencing effects from human trials that are not likely provided by the specific device, as well as supporting the claimed effect based on a sound research.
- In non-medical environment (e.g., at home), safety should also be standard. The published effects of tES on circumscribed cognitive processes can vary, which in some cases is attributable to nuanced differences in protocols (e.g., dose, task difficulty, subject state and prior training). If a given ‘home-applied’ protocol has not been formally tested, and many sensitive parameters not carefully reproduced, stimulation results are unpredictable. If a self-directed device and a self-controlled protocol (the subject decides for what kind of application, using which montage and stimulation duration) do not meaningfully copy the factors tested in human trials (e.g., the inclusions/exclusion criteria, the tested population or dose of the stimulation) then the outcomes of such stimulation may not be the same as in those trials. This means that such devices cannot readily claim the benefits of those trials.
- Regardless of intended use, we recommend that self-directed non-invasive brain stimulation devices be designed, produced, and distributed following engineering risk-management procedures, for example as outlined in the LOTES-2017 (Bikson et al., 2018) industry guidance and/or mandated by regulatory agencies such as the revised EU CE guidance. We do not suggest that all consumer brain stimulation devices must be regulated fully as medical devices and indeed such a recommendation cannot be easily implemented since many government agencies tasked with regulating medical devices do not explicitly regulate”wellness-use” devices.
- We also recommend continuous refinements of education and certification programs for researchers and clinicians employing brain stimulation methods worldwide, including workshops and education material, etc. This should address the potential irresponsible and unsafe use of NIBS methods, and the establishment of requirements regarding who can use these methods and under which circumstances, including ethical governance.

## Financial support and conflict of interest

ML and AA are supported by the Volkswagen Foundation German 2018 – Israeli Cooperation in Biological and Life Sciences, Medicine [number A128416]. AA is additionally supported by the BMBF (Stimcode, 01FP2124B).

AF is supported by the Deutsche Forschungsgemeinschaft (327654276 – SFB 1315 to AF).

BL is supported by the NIMH Intramural Research Program (ZIAMH002955).

VM acknowledges the financial support by STIPED funding from the European Union’s Horizon 2020 research and innovation programme (no. 731827).

GV acknowledges the support of Department of Biotechnology (DBT) – Wellcome Trust India Alliance (IA/CRC/19/1/610005) and Department of Biotechnology, Government of India (BT/HRD-NBA-NWB/38/2019-20(6)).

ARB receives scholarships and support from FAPESP (18/21722-8, 19/06009-6), the Brazilian National Council of Scientific Development (CNPq-1B), University of São Paulo Medical School (PIPA-FMUSP), and the UK Academy of Medical Sciences (Newton Advanced Fellowship), and the International Health Cohort Consortium (IHCC). ARB has equity in Flow Neuroscience, and receives in-kind support from Soterix Medical, MagVenture, Flow Neuroscience, Neurosoft, and NeuroConn.

The City University of New York holds patents on brain stimulation with MB as inventor. MB has equity in Soterix Medical Inc. MB consults, received grants, assigned inventions, and/or serves on the SAB of SafeToddles, Boston Scientific, GlaxoSmithKline, Biovisics, Mecta, Lumenis, Halo Neuroscience, Google-X, i-Lumen, Humm, Allergan (Abbvie).

CSH holds a patent on brain stimulation and was funded by the Deutsche Forschungsgemeinschaft (DFG, German Research Foundation) under Germany’s Excellence Strategy – EXC 2177/1 – Project ID 390895286.

CL is supported by the Swiss National Science Foundation (PZ00P3_179795). She is a member of the Scientific Advisory Board of Emma Sleep GmbH.

C Loo and her research program receive competitive grant funding from the Australian National Health and Medical Research Council.

FF and her research program are partially supported by the Italian National Institute of Health, Grant number GR-2016-02361802.

MH is supported by the NINDS Intramural Research Program. He is an inventor of patents held by NIH for an immunotoxin for the treatment of focal movement disorders and the H-coil for magnetic stimulation; in relation to the latter, he has received license fee payments from the NIH (from Brainsway). He is on the Medical Advisory Boards of CALA Health and Brainsway (both unpaid positions). He is on the Editorial Board of approximately 15 journals and receives royalties and/or honoraria from publishing from Cambridge University Press, Oxford University Press, Springer, Wiley, Wolters Kluwer, and Elsevier. He has research grants from Medtronic, Inc. for a study of DBS for dystonia and CALA Health for studies of a device to suppress tremor.

MAN is supported by Deutsche Forschungsgemeinschaft (DFG) – Projektnummer 316803389 – SFB 1280 “Funded by the Deutsche Forschungsgemeinschaft (DFG, German Research Foundation) – Projektnummer 316803389 – SFB 1280, the German Federal Ministry of Education and Research (BMBF, GCBS grant 01EE1501), and the EU (Neurotwin, H2020, grant 101017716).

NW is supported by the Swiss National Science Foundation (320030_175616) and the National Research Foundation, Prime Minister’s Office, Singapore under its Campus for Research Excellence and Technological Enterprise (CREATE) programme (FHT).

YU is supported by research grants from the Research Project Grant-in-aid for Scientific Research from the Ministry of Education, Culture, Sports, Science and Technology (grants number: 18K10821, 10H01091, 20K07866).

UZ received grants from the German Ministry of Education and Research (BMBF), European Research Council (ERC), German Research Foundation (DFG), Janssen Pharmaceuticals NV and Takeda Pharmaceutical Company Ltd., and consulting fees from Bayer Vital GmbH, Pfizer GmbH and CorTec GmbH, all not related to this work.

RH is currently supported by research grants from the NIH (R01AG059763) and U.S. Department of Defense (PR191513). He has received fees as a speaker by Starfish Neuroscience and Alexion Pharmaceuticals. None of these sources of support are related to this work.

RCK has equity in Cognite Neurothechnology, has a chief role in it, he is a member of the Scientific Advisory Board of Neuroelectrics and Innosphere.

WP is currently supported by a research grant of the EU Joint Programme – Neurodegenerative Disease Research (*JPND*).
